# Sphingomyelin Liposomes Containing Soluble *Leishmania major* antigens Induced Strong Th2 Immune Response in BALB/c Mice

**Published:** 2013-09

**Authors:** Omid Chavoshian, Nazanin Biari, Ali Badiee, Ali Khamesipour, Azam Abbasi, Zahra Saberi, Seyed Amir Jalali, Mahmoud Reza Jaafari

**Affiliations:** 1 **Nanotechnology Research Center, School of Pharmacy, Mashhad, University of Medical Sciences, Mashhad, Iran**; 2 **Biotechnology Research Center, School of Pharmacy, Mashhad, University of Medical Sciences, Mashhad, Iran**; 3 **Center for Research and Training in Skin Diseases and Leprosy, Tehran University of Medical Sciences, Tehran, Iran**

**Keywords:** Leishmaniasis, Liposome, Vaccine

## Abstract

***Objective(s):*** Soluble *Leishmania* antigens (SLA) provide suitable protection against leishmaniasis in murine model when delivered by an appropriate delivery system. Liposomes have been shown to be suitable vaccine delivery systems against leishmaniasis, however, the phospholipase-A (PLA) activity of SLA is a drawback to prepare a stable liposomal SLA. One strategy to overcome this problem might be using a lipid which is resistant to PLA activity of SLA such as sphingomyelin (SM). The aim of this study was to evaluate the effect of stable SM liposomes containing SLA on the immune response induced against leishmaniasis in BALB/c mice .

***Materials and Methods:*** BALB/c mice were immunized subcutaneously, three times with 2-week intervals, with SLA, SM-liposome-SLA, empty liposome or buffer. As criteria for protection, footpads swelling at the site of challenge and foot parasite loads were assessed. The immune responses were also evaluated by determination of IgG subtypes and the level of IFN-γ and IL-4 in cultured splenocytes.

***Results:*** The group of mice receiving SM-liposome-SLA, showed a significant large footpad swelling, higher parasite burden in foot and higher IL-4 level compared to the group immunized with buffer. In terms of IgG and IgG isotypes, there was no significant difference between the mice receiving SM-liposome-SLA and the mice that received buffer. Moreover, the immune response induced by SM-liposome-SLA showed no significant difference compared with the one caused by SLA alone.

***Conclusion:*** It is concluded that SM-liposome-SLA is not an appropriate strategy to induce Th1 immune response and protect the mice against Leishmaniasis; however, SM-liposomes could be suitable vaccine delivery systems when a Th2 response is needed.

## Introduction

*Leishmania* infection causes a group of diseases ranging from a self-healing cutaneous lesion to potentially fatal visceral form of disease, known as Kaka-azar. Control strategies are not always successful, available drugs need multiple injections and practically, show - limited efficacy in some endemic areas (1-3). For centuries, it was well known that long lasting protection induces upon recovery from cutaneous leishmaniasis (CL) caused by natural infection or leishmanization (4). Attempts to develop vaccines against leishmaniasis resulted in identifying numerous candidate antigens, but only first generation vaccines consisting of parasite fractions or whole killed *Leishmania* with or without adjuvant reached phase 3 clinical trials (4-7). However, the results were not conclusive in some trials and in general, the efficacy was limited mainly due to lack of a suitable adjuvant or delivery systems (8). Preparation of liposomes containing soluble *Leishmania* antigens (SLA) has attracted some researchers to produce a vaccine against the parasitic disease because of advantages of the first generation anti-*Leishmania* vaccines (9-10). SLA is a complex mixture of soluble antigens derived from whole* Leishmania *promastigotes (11) which induces protection against leishmaniasis in murine model if delivered with an appropriate adjuvant and/or delivery systems (9, 12). Liposomes are lipid-bilayer membranes capable of encapsulating antigens which act as efficient slow-releasing antigen delivery vehicle with depository effects. Liposomes have emerged as promising adjuvant system with low toxicity which protect antigens from damages and enhance uptake and presentation of encapsulated antigens by antigen presenting cells through MHC-I or MHC-II pathways (13).

One of the commonly used phospholipids to prepare liposomes is egg sphingomyelin (SM) which is primarily composed of saturated acyl chains and contains a single trans double bond in the sphingosine backbone. Sphingolipids exist especially in the plasma membrane and related cell membranes such as Golgi membranes and lysosomes of eukaryotic cells (14). Moreover, SM is able to form strong intermolecular hydrogen bonds with neighboring cholesterol (Chol) molecules to provide a very rigid membrane which is relatively impermeable compared with other lipid compositions (15). SM/Chol liposomes containing drugs such as vincristine and ciprofloxacin have been shown to have longer circulation times and more drug retention properties *in vitro* and *in vivo* compared with other liposomes (15). Inclusion of SM in the liposomal membranes with cholesterol enhances liposomal stability greatly (16). It was shown that SM/Chol formulation of vinorelbine is pharmaceutically stable for up to 1 year at 4-8°C (15).

The result of a recent study by the same research team (manuscript in preparation), revealed that SLA prepared from *Leishmania major* promastigotes has a phospholipase A (PLA) enzyme which hydrolyses egg phosphatidylcholine (EPC) in liposomal bilayer and causes instability and disruption of liposomes. However, it was considered that SLA causes no hydrolysis on liposomes consisting of SM due to the absence of carboxyl ester bond in its chemical structure. Hence, SM is used in this study to produce a stable liposomal SLA due to resistance to PLA activity.

Utilized to immunize BALB/c mice, the extent of protection and the type of immune response generated were studied in immunized mice compared with the control groups which received either HEPES-sucrose buffer or SLA alone.

## Materials and Methods

*Animals, parasites and SLA*

Female BALB/c mice, 6–8 weeks old, were purchased from Pasteur Institute (Tehran, Iran). The mice were maintained in animal house of Pharmaceutical Research Center and fed with tap water and laboratory pellet chow (Khorassan Javane Co., Mashhad, Iran). Animals were housed in a colony room 12/12 hr light/dark cycle at 21°C with free access to water and food. Experiments were carried out according to Mashhad University of Medical Sciences Ethical Committee Acts.

*Leishmania major* strain (MRHO/IR/75/ER) used in this experiment is the one used to prepare experimental *Leishmania* vaccine, leishmanin, and leishmanization (8, 17).

SLA preparation was carried out using the protocol developed by Scott *et al *(11) with minor modifications. Briefly, the parasites were harvested at stationary phase and washed 4 times using HEPES-sucrose buffer (10 mM, 10% w/v, pH= 7.4). Then, the number of promastigotes was adjusted to 1.2 × 10^9^/ml in buffer solution containing enzyme inhibitor cocktail, 50 µl/ml (Sigma, St. Louis, USA). Then, the parasites were lysed using freeze-thaw method followed by probe sonication in an ice bath. The supernatant of centrifuged lysate parasites was collected, dialyzed against buffer solution, sterilized using a 0.22 µm membrane and stored at -70°C until use. The protein concentration of the preparation was determined using BCA protein assay method (Thermo Scientific, USA).

*Liposomes preparation and characterization*

Liposomes containing SLA were prepared using detergent removal method as previously described (18). Briefly, the lipid phase consisting of sphingomyelin (13 µmol/ml; Avanti Polar lipids, USA) and cholesterol (1 µmol/ml; Avanti Polar lipids, USA) was dissolved in chloroform in a sterile tube. The solvent was then removed using rotary evaporator (Hettich, Germany) resulting in deposition of a thin lipid film on the tube’s wall. The lipid film was then freeze–dried (TAITEC, Japan) overnight to ensure complete removal of the solvent. The film was dissolved in HEPES-sucrose buffer (10 mM, 10% w/v, pH 7.4) containing C_12_E_8 _(100 mM; Sigma, St. Louis, USA) and SLA (1 mg/ml). The detergent-solubilized phospholipids and SLA were mixed and sonicated for 5 min to yield a clear mixed micellar colloid. Liposomes were then formed by detergent removal using SM2 Bio-Beads (BioRad, USA) according to the manufacturer’s instructions.

Particle size analyzer (Nano-ZS, Malvern,UK) was used to estimate the mean diameter and zeta potential of the liposomes. The concentration of SLA encapsulated in liposomes was determined using BCA protein assay kit (Thermo Scientific, USA).

*SDS-PAGE analysis of SLA and liposomal SLA*

Analytical SDS-PAGE was carried out to characterize and estimate qualitatively the concentration of SLA encapsulated in the prepared Lip-SLA. The gel consisted of running gel (10.22%, w/v, acrylamide) and stacking gel (4.78%, w/v, acrylamide) at the thickness of 1 mm. The electrophoresis buffer was 25 mM Tris, 192 mM glycine, 0.1% SDS, pH 8.3. Electrophoresis was carried out at 140 V constant voltages for 45 min and after electrophoresis; gels were stained using silver for protein detection.

*Immunization of BAlB/c mice*

Different groups of mice (ten mice per group) were already subcutaneously (SC) immunized three times in 2-week intervals with one of the following formulations: Lip-SLA (50 µg SLA/50 µl liposome/mouse), Empty Lip (50 µl empty liposome/mouse), SLA (50 µg SLA/50 µl /mouse), buffer (HEPES 10 mM, sucrose 10% w/v, pH 7.4) alone.

*Challenge with L. major promastigotes*

The immunized mice (five mice per group) were challenged SC in the left footpad with 1×10^6^ *L. major* promastigotes harvested at stationary phase in 50 µl volume, 2 weeks after the last booster injection. Lesion development was recorded in each mouse by measuring of footpad swelling using a metric caliper (Mitutoyo Measuring Instruments, Japan). Grading of lesion size was done by subtracting the thickness of the uninfected contralateral footpad from that of the infected one.

*Quantitative parasite burden in foot*

The number of viable *L. major* parasites in the foot of mice was estimated using limiting dilution assay method as described previously (19). Briefly, the mice were sacriﬁced at week 7 post-challenge; the feet were aseptically removed and homogenized in RPMI 1640 supplemented with 10% v/v heat inactivated FCS (Eurobio, France), 2 mM glutamine, 100 U/ml of penicillin and 100 µg/mL of streptomycin sulfate (RPMI-FCS). The homogenate was diluted with the media in 8 serial 10-fold dilutions and then was placed in each well of ﬂat-bottom 96-well microtiter plates (Nunc, Denmark) containing solid layer of rabbit blood agar in tetraplicate and incubated at 26 ± 1°C for 7-10 days. The number of viable parasite per foot was determined by ELIDA software, a statistical method for limiting dilution assay (20).

*Antibody isotype assay*

Blood samples were collected from mice before and at week 7 after challenge and the sera were isolated and kept at -20°C until being used to assess anti-SLA IgG total, IgG1 and IgG2a antibodies using ELISA method as described before (19). Briefly, 96-well micro titer plates (Nunc, Denmark) were coated with 50 µl of 10 µg/ml of SLA in PBS buffer (0.01M, pH= 7.3) overnight at 4°C. Plates were washed and then blocked by adding 200 µl per well of 1% of bovine serum albumin in PBS-Tween 20 and incubated at 37°C for 1 hr. Serum samples were diluted to 1:200, 1:2,000 or 1:20,000 with PBS–Tween and applied to the plates. The plates were then treated with HRP-rabbit anti-mouse IgG isotype according to the manufacturer’s instructions (Invitrogen Inc., USA). Optical density (OD) was determined at 450 nm using 630 nm as the reference wavelength.

*In vitro cytokine production by splenocytes*

ELISPOT assay was performed using mouse ELISPOT kits from U-cytech (Utrecht, The Netherlands) as directed by the supplier. Briefly, three mice from each group at week 2 after the last booster injection (before challenge) were sacriﬁced and their splenocytes were isolated and restimulated *in vitro* by either mitogen Concanavalin A (Con A) as a positive control or SLA as a recalled antigen. ELISPOT plates were coated with anti-IL-4 or anti-IFN-γ antibodies and incubated at 4°C overnight. The splenocytes (5 × 10^5^ cells/well) were then cultured in triplicate in a final volume of 200 μl with DMEM only (as background responses), medium containing Con A (as positive controls), or with medium containing 10 μg/ml of SLA in the pre-coated plates. After incubation (37°C, 5% CO_2_) of 24 hr (for IFN-γ assay) or 48 hr (for IL-4 assay), spot counting was done using a Kodak 1D software package (Version 3.5, Eastman Kodak, Rochester, New York). The mean number of spots ± SD in triplicate wells was calculated and expressed as spot-forming units (SFU) per 10^5^ splenocytes. 

*Statistical analysis*

One-way ANOVA statistical test was used to assess the significance of the differences among various groups. In the case of signiﬁcant F value, Tukey–Kramer multiple comparisons test was carried out as a post-test to compare the means in different groups of mice. Results with *P* < 0.05 were considered as statistically signiﬁcant.

## Results

*Liposome characterization*

**Figure 1:**
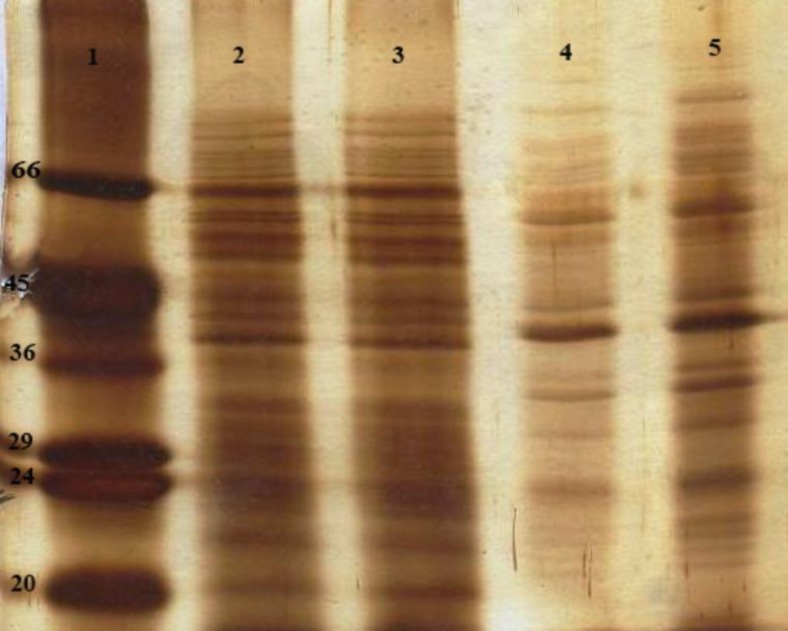
SDS-PAGE analysis of SLA alone and liposomal SLA. Lane 1, low-range protein standard (Sigma, USA); Lane 2, SLA (5 µg); Lane 3, SLA (10 µg); Lane 4, liposomal SLA (2.5 µg); Lane 5, liposomal SLA (5 µg

The mean diameter of Lip-SLA and Empty Lip formulations was 154.85 ± 7.68 and 116.86 ± 13.89 nm (n=3), and the zeta potentials were -25.63 mV and -27.43 mV, respectively. Characterization of SLA and liposomal SLA were performed by SDS-PAGE electrophoresis (Figure 1). SDS-PAGE analysis of SLA revealed several protein bands with different ranges of molecular weight. Also, the SDS-PAGE analysis of liposomal SLA revealed bands similar to free SLA. The calculated concentration of SLA and Lip-SLA in final formulations was 1 µg/ µl.

**Figure 2:**
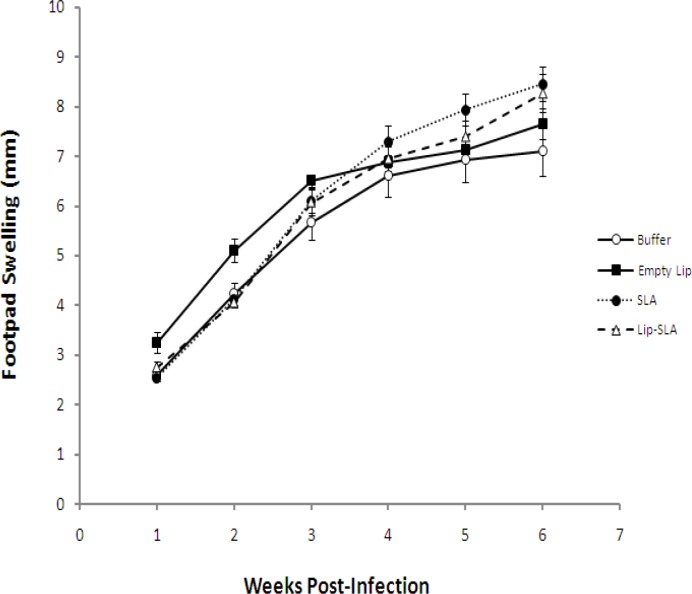
Footpad swelling in BALB/c mice immunized SC, three times with 2-week interval, with Lip-SLA, SLA, Empty Lip or buffer after challenge with 10^6^ virulent *Leishmania major* promastigotes in leftfootpads. The footpad thickness was measured weekly, for 6 weeks. Each point represents the average increase in footpad thickness ± SEM (n = 5)

*Challenge results*

Lesion development was monitored by weekly measurement of footpad thickness (Figure 2). The lesion size progressed at a rapid rate in mice immunized with SLA alone, Empty Lip, Lip-SLA, or buffer at week 2, 3 or 4 after challenge. There was no significant difference among lesions size of mice immunized with Empty Lip, SLA alone, Lip-SLA, or buffer at all weeks. Footpad thickness in all groups was progressed continuously and no protection was observed in any group. In all groups, the footpad swelling reached a plateau after 6 weeks but the disease progressed by metastasis to other organs.

*Parasite burden in foot*

**Figure 3:**
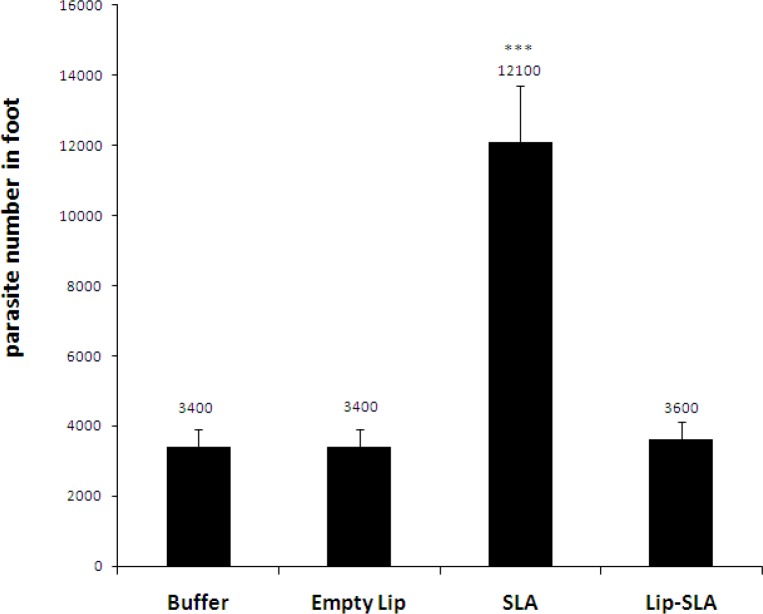
Foot parasite burden in BALB/c mice immunized SC, 3 times with 2-week interval, with Lip-SLA, SLA, Empty Lip or buffer after challenge with *Leishmania major* promastigotes. A limiting dilution analysis was performed at week 7 after the challenge on the foot of individual mice and cultured in tetraplicate in a serial of 8-fold dilutions. The number of viable parasite per foot was determined using ELIDA software. The bar represents the average score ± SEM (n = 3). (***) *P*< 0.001 is observed when the mice immunized with SLA are compared with the mice received Empty Lip, Lip-SLA or buffer

The number of viable *L. major* was determined in the infected foot of different groups of mice at week 7 after challenge (Figure 3). All groups of vaccinated mice showed live parasites in their foot. The number of parasites in the foot of mice immunized with SLA was more than the groups received Lip-SLA, Empty Lip or buffer (*P* <0.001).

*Antibody response*

**Figure 4:**
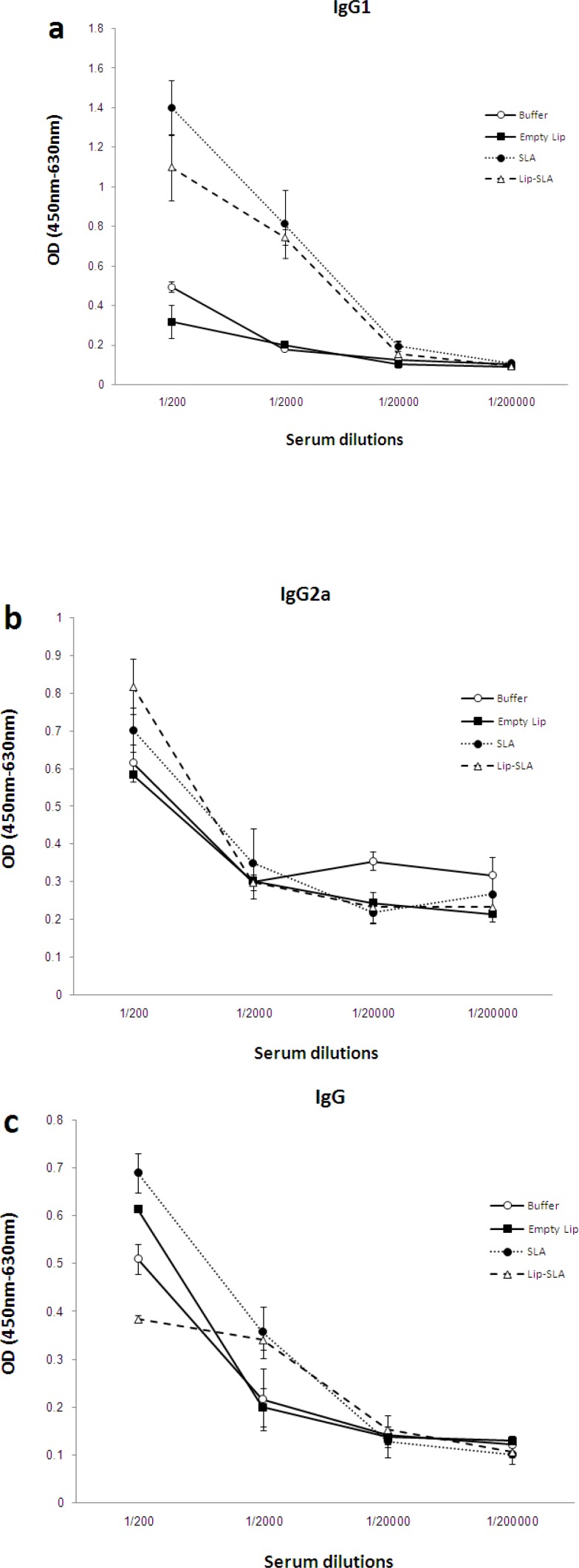
Levels of anti-SLA IgG1 (a), IgG2a (b), and IgG (c) in pooled sera of BALB/c mice immunized SC, three times with 2-week intervals, with Lip-SLA, SLA, Empty Lip or buffer. Blood samples were collected from the mice 2 weeks after the last booster. The anti-SLA IgG1, IgG2a and IgG levels were assessed using ELISA method. The assays were performed in triplicate at 200, 2,000, 20,000, or 200,000-fold dilution for each serum sample. Values are mean ± SD

**Figure 5:**
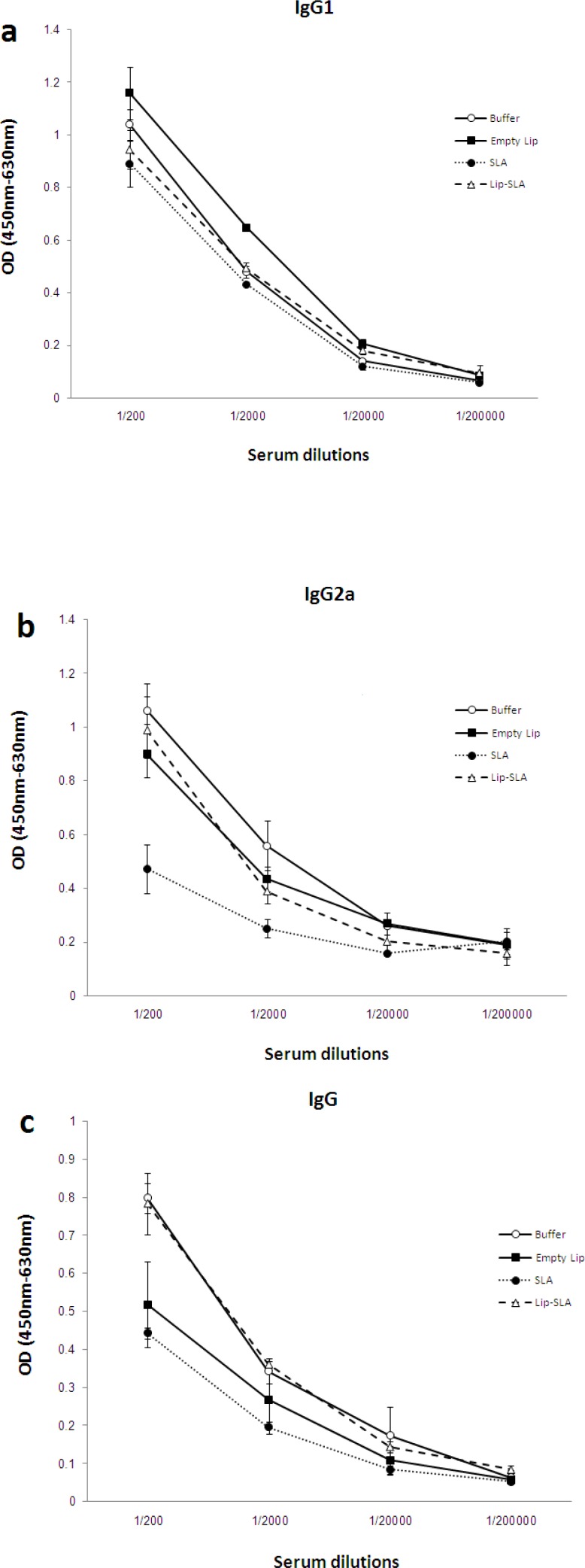
Levels of anti-SLA IgG1 (a), IgG2a (b), and IgG (c) in pooled sera of BALB/c mice immunized SC, three times in 2-week intervals, with Lip-SLA, SLA, Empty Lip or buffer. Blood samples were collected from the mice at week 7 after challenge. The anti-SLA IgG1, IgG2a and IgG levels were assessed using ELISA method. The assays were performed in triplicate using 200, 2,000, 20,000, or 200,000-fold dilution for each serum sample. Values are mean ± SD

In order to determine the type of generated immune response, the anti-SLA IgG antibodies and IgG1, IgG2a subclasses were titrated before (Figs. 4A, 4B and 4C) and after (Figures 5A, 5B and 5C) the challenge. 

Generally, there was no significant difference in the level of IgG and IgG isotypes (IgG1, IgG2a) in serum dilution of 1:20,000 or more in immunized mice (before or after challenge). The sera of pre-challenged mice immunized with Lip-SLA or SLA showed significantly (*P* < 0.05) higher levels of IgG1 antibodies compared to the other groups particularly in serum dilutions of 1:200 and 1:2000. However, after challenge, there was no significant difference between the level of IgG1 in these two mentioned immunized groups compared with the control groups (buffer or Empty Lip). 

In terms of IgG2a, there was no significant difference between mice in different groups before challenge (*P* > 0.05). On the contrary, the sera of post-challenged mice immunized with SLA showed significantly (*P* < 0.05) lower levels of IgG2a antibodies compared to the other groups in serum dilutions of 1:200 or 1:2000 and there was no significant difference in the level of IgG2a in mice which received buffer, empty Lip, or Lip-SLA after challenge.

**Table1:** The ratio of IgG2a/IgG1 before challenge

Groups	Serum dilution			
	1/200	1/2,000	1/20,000	1/200,000
				
Buffer	1.25	1.68	2.82	3.09
Empty Lip	1.14	1.51	2.32	2.38
SLA	0.50	0.43	1.13	2.52
Lip-SLA	0.74	0.40	1.52	2.55

The sera of pre-challenged mice immunized with SLA or Empty Lip showed signiﬁcantly (*P* < 0.05) higher levels of IgG antibodies compared to the other groups, particularly in serum dilution of 1:200 or 1:2000 but there was no significant difference between the two groups (*P* >0.05). In the sera collected after challenge in the group of mice that received buffer or Lip-SLA, signiﬁcantly (*P* < 0.05) higher levels of IgG antibodies were recorded compared to the other groups, particularly in serum dilutions of 1:200 or 1:2000. 

Interestingly, the highest ratio of IgG2a/IgG1 belonged to the sera of mice immunized with buffer compared with the other groups before and after the challenge (Table 1). 

*In vitro cytokine production by splenocytes*

**Figure 6:**
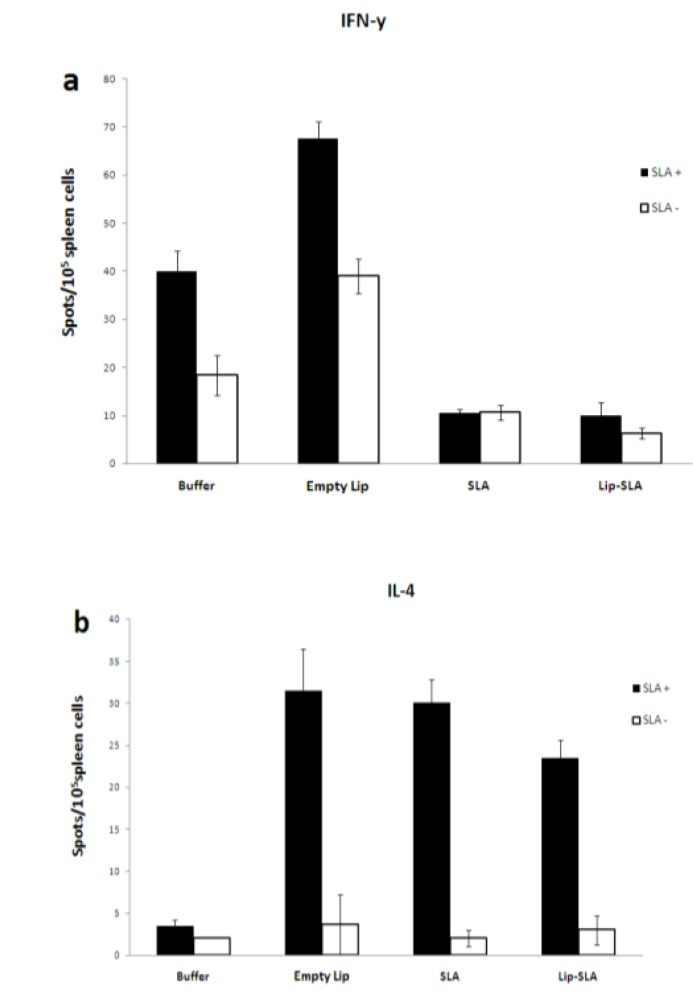
Cytokine levels in immunized mice at week 2 after the last booster injection. Mononuclear splenocytes were cultured in the presence of SLA (10 µg/ml) and the level of IFN-γ (a) or IL-4 (b) in the culture supernatants were detected using ElISPOT method. Results are shown as Mean ± SEM (n = 3

The results of ELISPOT assays showed that splenocytes isolated from the mice immunized with Empty Lip, SLA or Lip-SLA released a significantly (*P*< 0.05) higher amounts of IL-4 (Figure 6, B), in comparison with the group of mice immunized with buffer. There was no significant difference between the groups of mice which received SLA or Lip-SLA. Also, the results showed splenocytes isolated from the group of mice immunized with SLA or Lip-SLA released a significantly (*P* < 0.05) lower amounts of IFN-γ (Figure 6, A), in comparison with the groups of mice received buffer or empty Lip (*P* < 0.05). There was no significant difference between the groups of mice immunized with Lip-SLA or SLA or Empty Lip, in terms of IL-4 secretion (Figure 6, B).

## Discussion

In the present study, SLA was used as a model of crude first generation vaccine based on the previous studies that showed cocktail vaccines may provide a wider range of potentially protective epitopes taking advantage of these antigens that induce the required immunity (mainly CD4^+^ and CD8^+^ IFNγ-mediated responses) (21). A specific polypeptide might be a strong immunogen when used as a cocktail, although individually it might be a weak immunogen or induce only partial protection (22). Moreover, SLA’s components of *L. donovani* might be a candidate vaccine for future studies if used in liposomal form (23). SLA included most of the *Leishmania* soluble antigens and induced more protection than recombinant antigens such as gp63 and LAg when was used in liposomal form (23). In the current study, immunization with SLA alone induced no protection in BALB/c mice based on the data from footpad swelling and parasite burden in foot. The ratio of IgG2a/IgG1 (a marker for the induction of Th1- like response) in mice immunized with SLA was less than 0.6 (in serum dilution of 1:200 or 1:2000) and also the high level of IL-4 production was assessed in their splenocytes which confirmed the footpad swelling and parasite burden results. Exacerbation by SLA in the absence of any Th_1_-promoting adjuvant is not surprising in BALB/c mice due to the tendency to develop a Th2 type of response. The co-existence of Th1/Th2 responses with SLA immunization is consistent with the previous studies (24-25).

The role of lipid compositions in liposome formulation in shifting the generated immune response was studied before considering different phospholipids against leishmaniasis (26). The results indicated that the generated immune responses in mice was influenced by the bilayer composition of liposomes, so that mice immunized with liposomes consisting of EPC induced a Th2 type of immune response while liposome consisting of DPPC or DSPC induced Th_1_ type of immune response. It seems that liposomes prepared with higher phase transition temperature (T_m_) phospholipids are suitable formulation to induce Th_1_ type of immune response and protection, however, the existence of phospho-

lipase enzyme in *Leishmania *spp. (27), particularly first generation vaccines which generally consisting of killed *Leishmania* or parasite fractions such as SLA seems to be a limitation to prepare an effective anti-*Leishmani*a liposomal vaccine. Hence, in this study, SM was used to prepare a stable liposome formulation containing SLA. SM has no Acyl bond in its structure and when SM was used as an Acyl-bond-free lipid to prepare liposome containing SLA, there was no sign of SM hydrolysis in chromatogram and dynamic light scattering (DLS) results (data not shown). 

**Table 2:** The ratio of IgG2a/IgG1 at week 7 after challenge

Groups	Serum dilution			
	1/200	1/2,000	1/20,000	1/200,000
				
Buffer	1.02	1.17	1.84	2.94
Empty lip	0.78	0.67	1.30	2.22
SLA	0.53	0.58	1.33	3.50
Lip-SLA	1.05	0.79	1.12	1.67

In this study, liposome consisting of SM was used as a stable antigen delivery system because of its resistance to PLA activity of SLA and more importantly, its adjuvanticity was also evaluated for the first time against leishmaniasis. SM, a major membrane sphingolipid, is the precursor of the bioactive products (28). It was proved that sphingolipid metabolism is a dynamic process and sphingolipid metabolites such as ceramide, sphingosine and sphingosine 1-phosphate (S1P) are now recognized as messengers playing essential roles in cell growth and survival (29). It is clear now that signal transduction through SM and ceramide strongly affect the immune response generation either directly through cell signaling events or indirectly through cytokines produced by other cells as the result of signaling through the SM pathway (30). 

To assess the protection rate, footpad swelling induced by *L. major* challenge and foot parasite burden were checked in different groups of mice. The results showed that there was no significant difference (*P *< 0.05) between the size of the lesion in groups of mice immunized with empty Lip, SLA alone or Lip-SLA and the lesions size were larger than lesion size of mice received buffer but it was not significant. Therefore, the SM liposome was not able to protect mice against challenge with *L. major* infection. The results of foot parasite burden also confirmed this issue. In terms of IgG subclasses, encapsulation of SLA into SM-liposome resulted in no significant increase in production of IgG2a antibodies and IgG2a/IgG1 ratio (Table 2). The results of cytokine assay also showed that splenocytes isolated from the mice immunized with SLA or Lip-SLA released significantly (*P* < 0.05) higher amounts of IL-4 and lower amounts of IFN-γ in comparison with the group of mice immunized with buffer (*P* < 0.05). The generated results might be due to the complex immunomodulatory properties of SM. The SM cycle products were shown to affect the CD3, CD4, CD8, CD45, and other T lymphocyte surface antigen expression (31). It is now becoming increasingly apparent that sphingolipids can be intimately involved in inflammation (32). It is demonstrated that ceramide itself activates inflammatory pathways via NF-κB gene transcription. Ceramide also up-regulates another family of transcription factors closely associated with inflammation. CCAAT/enhancer binding proteins (c/EBP) induce gene expression of several inflammatory proteins including TNF, IL-6, IL-8 and IL-1β (32). While ceramide is often antiproliferative and proapoptotic, S1P was shown to act as a second messenger in cellular proliferation and survival and in protection against ceramide-mediated apoptosis. Thus, it is suggested that the balance between these two sphingolipid messages may be an important factor determining survival or death of mammalian cells (29). So, identified or proposed roles of sphingolipids or SM cycle products vary and some of the sphingolipids such as ceramide and S1P showed opposing roles. Also, studies about the functions of these lipids are not without controversy (16). Therefore, it is difficult to predict whether these results are related to the immunomodulatory properties of SM or not. In fact, the current results showed that SM liposomes probably induced a Th_2_ type of immune response against leishmaniasis in BALB/c mice so that infection and lesions were exacerbated in the immunized group. 

## Conclusion

The current data point out that SLA in association with SM liposomes elicits no protective responses against murine model of leishmaniasis and liposomal SLA showed no significant differences with SLA alone in terms of protective efficacy. The current data revealed that SM-liposome-SLA is not an appropriate strategy to induce Th1 type of immune response and protect mice against Leishmaniasis; however, SM-liposomes could be suitable vaccine delivery system when a Th_2_ response is needed.
